# A historical review of HIV prevention and care initiatives in British Columbia, Canada: 1996-2015

**DOI:** 10.7448/IAS.20.1.21941

**Published:** 2017-09-19

**Authors:** Michelle Olding, Ben Enns, Dimitra Panagiotoglou, Jean Shoveller, P Richard Harrigan, Rolando Barrios, Thomas Kerr, Julio S. G. Montaner, Bohdan Nosyk

**Affiliations:** ^a^ BC Centre for Excellence in HIV/AIDS, St. Paul’s Hospital, Vancouver, BC, Canada; ^b^ School of Population and Public Health, University of British Columbia, Vancouver, BC, Canada; ^c^ Division of AIDS, Faculty of Medicine, University of British Columbia, Vancouver, BC, Canada; ^d^ Faculty of Health Sciences, Simon Fraser University, Burnaby, BC, Canada

**Keywords:** British Columbia, historical review, cascade of care, treatment as prevention, quality of care, harm reduction

## Abstract

**Introduction**: British Columbia has made significant progress in the treatment and prevention of HIV since 1996, when Highly Active Antiretroviral Therapy (HAART) became available. However, we currently lack a historical summary of HIV prevention and care interventions implemented in the province since the introduction of HAART and how they have shaped the HIV epidemic. Guided by a socio-ecological framework, we present a historical review of biomedical and health services, community and structural interventions implemented in British Columbia from 1996–2015 to prevent HIV transmission or otherwise enhance the cascade of HIV care.

**Methods**: We constructed a historical timeline of HIV interventions implemented in BC between 1996 and 2015 by reviewing publicly available reports, guidelines and other documents from provincial health agencies, community organizations and AIDS service organizations, and by conducting searches of peer-reviewed literature through PubMed and Ovid MEDLINE. We collected further programmatic information by administering a data collection form to representatives from BC’s regional health authorities and an umbrella agency representing 45 AIDS Service organizations. Using linked population-level health administrative data, we identified key phases of the HIV epidemic in British Columbia, as characterized by distinct changes in HIV incidence, HAART uptake and the provincial HIV response.

**Results and Discussion**: In total, we identified 175 HIV prevention and care interventions implemented in BC from 1996 to 2015. We identify and describe four phases in BC’s response to HIV/AIDS: the early HAART phase (1996–1999); the harm reduction and health service scale-up phase (2000–2005); the early Treatment as Prevention phase (2006–2009); and the STOP HIV/AIDS phase (2010-present). In doing so, we provide an overview of British Columbia’s universal and centralized HIV treatment system and detail the role of community-based and provincial stakeholders in advancing innovative prevention and harm reduction approaches, as well as “seek, test, treat and retain” strategies.

**Conclusions**: The review provides valuable insight into British Columbia’s HIV response, highlights emerging priorities, and may inform future efforts to evaluate the causal impact of interventions.

## Introduction

British Columbia (BC) has made substantial progress treating and preventing HIV since the infection emerged in the early 1980s. The introduction of highly active antiretroviral therapy (HAART) in 1996 heralded a turning point in BC’s HIV response, leading to dramatic declines in HIV-related morbidity and improvements in life expectancy [1]. The province continues to observe reductions in HIV transmission, with new cases falling from a high of 18.1 per 100,000 in 1995 to 5.1 per 100,000 in 2015 [1–3]. Model projections suggest that BC is on track to achieve 2020 targets set by the Joint United Nations Programme on HIV/AIDS (UNAIDS) of 90% of all People Living with HIV (PLHIV) diagnosed, 90% of those diagnosed on HAART and 90% of those on HAART virologically suppressed [4].

Given accumulating evidence that HAART prevents HIV transmission [1,5–9], a wide range of initiatives to expand HAART uptake likely contributed to declines in new cases [10]. The simultaneous expansion of harm reduction services, including syringe distribution, opioid agonist treatment, and supervised injection facilities had a profound impact on the epidemic by reducing HIV transmission through needle sharing [10]. Governmental, clinical, and community-based groups, including drug user organizations, have also undertaken prevention and care initiatives since the onset of HAART to facilitate access to HIV care and prevention [11,12]. However, given the diversity and scope of such initiatives, we currently lack a historical summary of these activities and their implications for the HIV epidemic.

This article provides a historical summary of HIV prevention and care initiatives implemented in BC since the introduction of HAART (1996–2015). We characterize and categorize interventions within a socio-ecological framework to highlight the biomedical and health services, community and structural factors shaping the HIV epidemic in BC. Categorizing and summarizing interventions into a single timeline provides scientific and health administrative communities with valuable insight into BC’s HIV response and may inform future efforts to evaluate the causal impact of interventions.

## Methods

We constructed a timeline of HIV prevention and care initiatives by reviewing publicly-available documents, guidelines and reports retrieved online from provincial health agencies, community organizations and AIDS service organizations (See Supplementary Appendix, Table 1). We conducted directed searches of the peer-reviewed literature on OVID Medline and PUBMED to retrieve English-language articles. Search words included subject k eywords (HIV diagnosis; sexually transmitted diseases; HIV prevention and control; HIV drug therapy; methadone; health knowledge, attitudes, practice; delivery of HIV care; antiretroviral therapy, highly active; patient compliance; medication adherence; needle exchange; needle syringe program; harm reduction; opioid substitution treatment; methadone; pilot project; condoms; family planning services; health behavior; sex education; sexual behavior) and a location term (BC).

Peer-reviewed and grey literature sources were included if they described or evaluated an HIV prevention or care intervention in BC during the study period (1996–2015). We defined HIV prevention and care interventions as any biomedical and health services, community level, or structural intervention implemented to reduce HIV transmission, improve quality of HIV care or otherwise enhance engagement of people living with HIV (PLHIV) along the HIV treatment cascade from diagnosis to viral suppression [13]. We collected programmatic information through personal communication with key informants from BC’s 5 regional health authorities (Table 1).

**Table 1.** Population size of British Columbia’s regional health authorities, 2015

Geographical region	Population size (2015)
Fraser Health Authority	1,733,902
Interior Health Authority	743,656
Northern Health Authority	288,399
Vancouver Coastal Health Authority	1,157,116
Vancouver Island Health Authority	767,505
British Columbia (total)	4,751,612

Sources: Population sizes from Statistics Canada and BC Ministry of Health [14].

Interventions were categorized by the cascade step addressed (*prevention of infection; diagnosis; linkage to care; retention in care; HAART uptake; HAART adherence; viral suppression*) and the socio-ecological level of influence at which they operated in preventing HIV or improving quality and reach of HIV care (*biomedical and health services, community, or structural*). Socio-ecological models have been previously developed to explain how factors at multiple levels interact to drive HIV transmission [15] and PLHIV engagement across the cascade of care [13,15–17]. The lead author constructed predetermined categories using existing socio-ecological models [13,18]. These categories were refined in collaboration with the senior author during early stages of analysis. The second author reviewed the first wave of coding and made modifications to ensure consistency in coding practices. The final list of coded interventions was reviewed and approved by all other co-authors.

Our socio-ecological framework situates interventions within three levels of influence: (1) *Biomedical and health services*, including delivery of biomedical interventions and health services to people living with and vulnerable to HIV; (2) *Community* *-level interventions*, including interventions to change beliefs, practices and social norms around HIV risk behaviours and health-seeking behaviours and; (3) *Structural interventions*, including legal, policy implementation, funding and other structural interventions shaping access to and the delivery of prevention, health care and social services.

We used population-level data from the “Seek and Treat for Optimal Prevention of HIV/AIDS” (STOP HIV/AIDS) cohort to construct figures depicting trends in the cascade of care and new HIV diagnoses from 1996 to 2015 (See Supplementary Appendix, Table 2 for definitions used for cascade steps) [19]. The STOP HIV/AIDS cohort is a census of all identified HIV positive individuals in BC from 1996 onward, constructed through provincial-level linkage of seven health administrative databases and disease registries, including the province-wide antiretroviral dispensation, virology and HIV testing registries, the Medical Services Plan database, the discharge abstract database, the BC PharmaNet database, and the BC Vital statistics database. Further details regarding the construction of the cohort and available databases are described in detail elsewhere [20].

To examine the socio-spatial uptake of HIV testing across BC Health Service Delivery Areas, we used BC Centre for Disease Control testing data to develop a heat map of HIV testing rates from 2009–2015 [21,22].

We constructed a figure depicting sterile syringe distribution and opioid agonist treatment enrolment using annual numbers of sterile syringe distributions reported by the BC Centre for Disease Control (available 2006–2013) [23] and number of opioid agonist patients reported by the Office of the Provincial Health Officer (available 1998–2014) [24,25].

These figures are not intended to demonstrate a causal relationship between specific initiatives and outcomes, but rather to provide context for changing trends in health service delivery and HIV epidemiology over the study period. Using this population level data, we aimed to identify and describe phases of the HIV epidemic in BC, as characterized by changes in new HIV cases, HAART uptake and the provincial HIV response.

## Results

**Figure 1.**
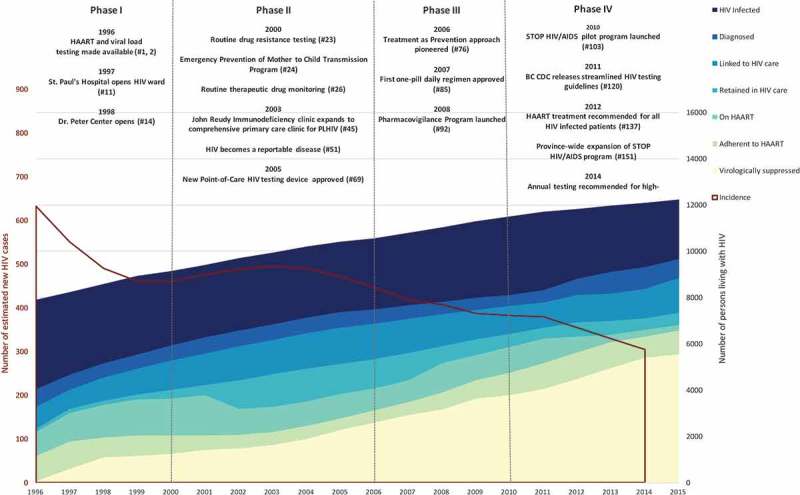
The cascade of HIV care, estimated new HIV cases, and key interventions in British Columbia: 1996–2015. Numbers in parentheses refer to chronologically numbered interventions in Supplementary Appendix, Table 3. HAART = highly active antiretroviral therapy. HIV incidence and prevalence data (HIV Infected) are based on estimates from the Public Health Agency of Canada (PHAC), available up until 2014.

**Figure 2.**
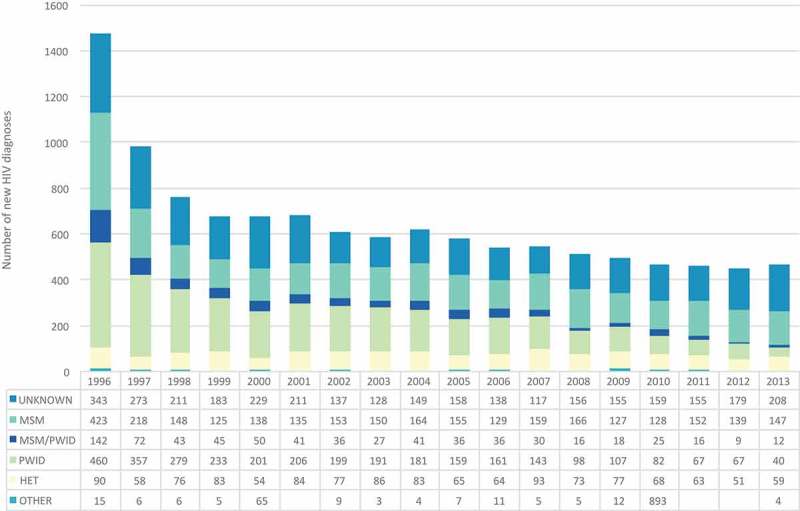
*New HIV diagnoses in British Columbia by exposure category, 1996–2014*. MSM: Men who have sex with men; PWID: people who inject drugs; HET: Heterosexuals.

Our grey-literature search retrieved therapeutic guidelines, programme reports, policy documents, newsletters and other documents described in Table 1 of the Supplementary Appendix. Our literature search, after removing duplicates and screening out irrelevant articles, retrieved 229 peer-reviewed articles for abstract review. Based on our review of these sources and personal communication with key informants, we identified 175 interventions implemented in BC from 1996 to 2015 (See Table 2 and Supplementary Appendix, Table 3). We identify and describe four phases of BC’s HIV epidemic below. Figure 1 provides a longitudinal perspective of population-level HIV incidence and HIV care cascade outcomes alongside selected interventions and policy changes during each phase of the HIV epidemic in BC. We present a breakdown of new HIV diagnoses by exposure category for available years of 1996–2013 (Figure 2).

**Table 2.** Chronologically numbered HIV prevention and care interventions in British Columbia, Canada (1996–2016), as detailed in the supplementary appendix

		Chronologically numbered HIV prevention and care interventions in British Columbia, as detailed in the supplementary appendix.			
		1996–1999	2000–2005	2006–2009	2010–2015
**Biomedical and Health Services** Delivery of biomedical interventions and health services to people living with and vulnerable to HIV	Antiretroviral drug development	1,9		85	
	HIV testing and screening technology	2	23, 63, 75	78, 83	113, 114, 140
	Therapeutic monitoring and surveillance		26	92	
	Harm reduction services	3, 4, 16	25, 28, 29, 30, 34, 35, 37, 40, 41, 42, 43, 48, 50, 54, 55, 66, 70, 71	84,87, 89	130, 132, 136
	Substance use treatment	3, 4	43, 56, 70		132
	Condom and safer sex supplies distribution		25, 50, 54, 61, 66	99	
	General HIV testing and counselling				129, 158, 165, 167
	Targeted HIV testing and counselling	5	49, 59	95	108, 113, 121, 122, 124, 125, 127, 128, 146, 154, 155, 160, 166, 168, 169
	Point-of-care HIV testing and counselling			95	105, 109, 118, 128, 146, 154
	HIV treatment services	10, 11, 14, 21, 22	32, 43, 45, 46, 52, 71	87, 90, 91, 94, 98,99	108, 126, 139, 147, 148, 157, 158, 160, 163, 164
	Case management services				130, 170
	Quality improvement initiatives				111
	Medication adherence support	21		88,96	117
	Patient alert systems			93	131, 150
**Community** Interventions to change beliefs, practices and social norms around HIV risk behaviours and health-seeking behaviours.	Peer education and outreach	6, 7,8, 12, 15, 17	23, 27, 30, 47, 60, 61, 62, 65, 67, 73, 74	87,88	105, 106, 115, 116, 121, 144, 145, 146, 149 156, 174
	Social marketing and public awareness campaigns	15	81		100,101,103, 133, 143,135, 161, 172, 173, 175
	Peer navigation	6, 17	31, 32	80, 87, 97	105, 113, 119, 132, 167, 170
	Supportive housing for PLHIV	14	58, 71		131, 170
	Food and nutrition programmes for PLHIV	19		82	
**Structural** Legal, policy implementation, funding and other structural interventions that shape access to and the delivery of prevention, health care and social services	Health system financing		53, 68	82	103, 151, 153
	Laws		51		136
	Policies	3, 4, 20	28, 34, 35, 52, 53, 54		103, 107, 119, 120, 137, 151
	Guidelines		36	77	152, 154

## The early HAART phase: 1996–1999

### Biomedical and health services

The beginning of the modern HAART era is commonly attributed to the 1996 discovery of triple combination antiretroviral therapy as a highly effective treatment for HIV [26–28]. HAART and viral load measurement were rapidly implemented as the standard of therapeutic care in BC, leading to unprecedented declines in AIDS cases, deaths and hospitalizations (Supplementary Appendix, Table 3, Interventions #1, 2) [29]. HAART has since been available free-of-charge to all medically-eligible British Columbians through the BC Centre for Excellence in HIV/AIDS (BC-CfE) Drug Treatment Program, which is centrally responsible for distributing and monitoring antiretrovirals according to IAS-USA consistent guidelines.

With effective treatment regimens identified, BC’s HIV response in the late 1990s was characterized by HAART scale up, facilitated by new provider-training programmes (#7, 18) and health services for PLHIV (#5,10,11,13 14, 21, 22). Many early HIV care and support services developed within Vancouver’s West End, a neighbourhood which experienced a highly concentrated burden of HIV and AIDS during the initial outbreak among MSM [30] .The first British Columbians to receive HAART were predominantly treated at the Immunodeficiency Clinic and a newly created AIDS ward located in Vancouver’s St. Paul’s hospital (#10). Specialty clinics launched during this time include Oak Tree Clinic (c.1994), an interdisciplinary specialized care and outreach programme for women, children and youth (#24, 153); the Dr. Peter Program (c.1998), a day health programme and 24-hour specialized care programme for PLHIV with complex needs (#14); and the Positive Outlook Program (c. 1997), an HIV care programme focused on culturally appropriate care for Indigenous PLHIV.(#10) Workshops developed by the BC Centre for Excellence in HIV/AIDS (BC-CfE) and BC Persons with AIDS Society were delivered to rural regions across the province starting in 1999 to disseminate latest information about HIV treatment options (#18).

The province experienced a second explosive outbreak in the mid-90s as new HIV diagnoses increased rapidly among people who inject drugs (PWID) and female sex workers (FWS) within Vancouver’s Downtown Eastside neighbourhood (Figure 2) [31,32]. A confluence of factors – including a network of high-density single room occupancy hotels, de-institutionalization of mental health services and shifts towards injectable cocaine use – contributed to the neighbourhood becoming a hub for drug-related harm and mortality [33,34]. High HIV transmission rates between 1996 and 1997 resulted in up to 40% of Vancouver’s estimated 15,000 PWID becoming HIV infected by the end of 1997 [31], prompting the Vancouver/Richmond Health Board to declare a public health emergency [35].

An outbreak investigation of this epidemic, initiated in 1995, eventually evolved into a prospective cohort study of Vancouver-based injection drug users known today as the Vancouver Injection Drug User Study (#5). In addition to collecting prospective data on drug use and behavioural trends among PWID, the study serves a critical public health function by providing regular HIV and HCV testing to PWID within the cohort, and disseminating public health information through peer workers. Early data from the cohort suggested Vancouver’s existing needle exchange programme, in operation since 1986, was not sufficient on its own to curb HIV transmission [36]. Many PWID continued to experience difficulty accessing syringes [36], indicating an urgent need for implementation and evaluation of additional strategies to address drug-related harms and their social determinants [31].

### Community

The HIV epidemic in Vancouver’s Downtown Eastside also served as a catalyst for drug user organizing and advocacy. In 1997, Downtown Eastside-based support and advocacy groups formed the Vancouver Area Network of Drug Users (VANDU), a drug-user run organization which aims to create a role for injection drug users in decision-making about the neighbourhood and programme delivery [37]. Community organizing and advocacy within BC’s MSM communities has also been central to BC’s response since the beginning of the epidemic. Organizations with roots in MSM communities, including AIDS Vancouver and the BC Persons with AIDS Society, continued to expand peer support and prevention programmes to address the epidemic among MSM (#15), while also providing wider reaching peer education and support services to PLHIV, including an HIV newsletter about treatment programmes (312) and a prison outreach programme (#7).

### Structural

**Figure 3.**
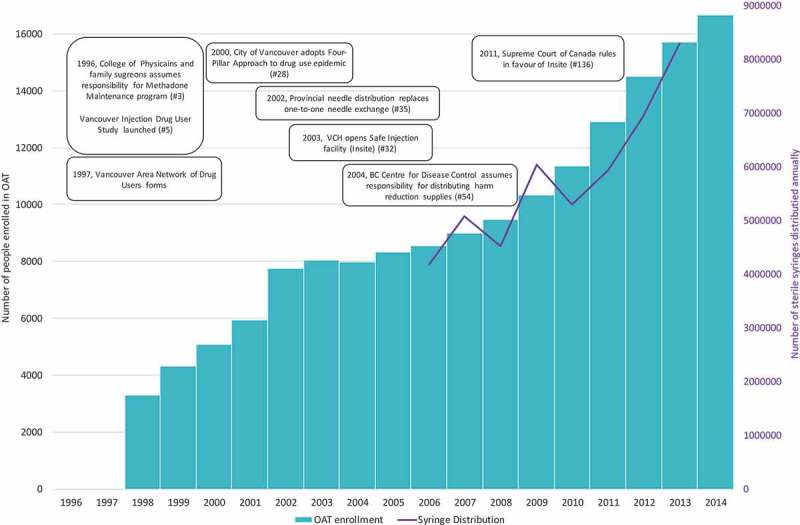
Opioid Agonist Treatment (OAT) enrolment and syringe distribution in British Columbia, 1996–2014. OAT: Opioid Agonist Treatment. VCH: Vancouver Coastal Health. IDU: Injection Drug User. Data Sources: BC Centre for Disease Control (syringe distribution) and the Office of the British Columbia Provincial Health Officer (OAT enrolment).

A policy change with considerable implications for the HIV epidemic was the 1996 federal government’s transfer of administrative responsibility for methadone maintenance treatment programmes to provincial medical licensing boards, including the College of Physicians and Surgeon of British Columbia (#3). The College subsequently relaxed constraints for physicians wishing to provide methadone, the first-line opioid agonist treatment for opioid dependence, and approved office and community-pharmacy based dispensation. These changes led to considerable growth in the number of clients accessing opioid agonist treatment each year with overall enrolment increasing from only 2,800 clients in 1996 to approximately 15,418 in 2014 (Figure 3) [25]. Given opioid agonist treatment has been associated with decreased needle-sharing [38], decreased HIV mortality[39] and improved HAART adherence [40], expanded access to opioid agonist treatment likely played a critical role in the decline of BC’s HIV epidemic [10,41].

## The harm reduction and health service scale-up phase: *2000–2005*

### Biomedical and health services

The early 2000s were characterized by a rebound in absolute numbers of new HIV cases, and stagnation in the proportion of diagnosed PLHIV receiving HAART (Figure 1). With only a limited set of antiretroviral drugs available and many clients having developed resistance through mono- or duo-therapy in the pre-HAART era, multi-drug resistance became a growing concern locally and internationally [42]. The introduction of routine drug resistance testing from 1999/2000 onward (#26) led to disconcerting findings that approximately 25% of treatment-exposed patients had multidrug resistant strains of HIV [43]. Poor tolerability and complex dosing requirements of HAART regimens likely contributed to sub-optimal adherence, which was in turn associated with multidrug resistance and treatment failure [44]. Baseline (pre-therapy) genotypic resistance testing, which was rare in 2000, became routinely conducted in BC by 2004/5 [45]. Laboratory research conducted at the BC-CfE during this time identified genetic factors pre-disposing individuals to HIV resistance, allowing for identification of more tailored and effective treatment regimens (#75).

A number of new health and social services opened in downtown Vancouver during the early 2000s (#33, 43), while core HIV care sites expanded to provide a more comprehensive range of services. These services included introduction of a perinatal HIV screening programme implemented in 27 hospitals to identify and treat pregnant women living with HIV(#24); expansion of the Dr. Peter Centre to become a stand-alone HIV care facility for people living with concurrent conditions (#14); and a 2003 re-design of The John Reudy Immunodeficiency Clinic, operating since 1986 at St. Paul’s Hospital, into an integrated, interdisciplinary primary and specialized care clinic for complex HIV cases (#50).

Harm reduction infrastructure expanded considerably at the turn of the millennium, reflecting a changing approach to drug use. After nearly a decade of focused advocacy from community groups such as VANDU, the City of Vancouver adopted policy in 2000 acknowledging harm reduction as a key pillar for addressing drug use alongside prevention, treatment and enforcement (#28) [46]. This “four pillar” policy called for decentralization of needle exchange services and the establishment of a supervised injection facility where people could inject illicitly-obtained drugs under supervision of health care staff (#48). Supervised injection facilities had been previously operated by the Dr. Peter Centre (#71) and drug user groups (#8, 73), however these sites were “unsanctioned” and subject to closure if and when police enforced federal drug laws. The first sanctioned supervised injection facility was realized in 2003, when Health Canada granted Vancouver Coastal Health permission to pilot and evaluate a supervised injection facility in the DTES. Research from this evaluation subsequently demonstrated effectiveness of the programme in reducing syringe sharing [47,48], reducing levels of public injecting in the immediate vicinity [49], reducing fatal overdoses [50], and increasing uptake of addiction treatment programmes [51] including referral to a medically-monitored detox unit opened onsite in 2004 (#56) [52].

Outside of Vancouver, non-profit agencies and health authorities faced the challenge of distributing harm reduction and other prevention services to rural regions, often with less health service infrastructure and restrictive local policies banning syringe exchange. Nonetheless, by 2010, fixed-site needle exchange programmes and mobile needle vans had been established by non-profit organizations in Vancouver, Victoria (#37), Fraser Valley (#66), Prince George (#29) and Kelowna (#16), with funding support from regional health authorities.

### Community

BC AIDS Services Organizations and community groups have a long history of providing peer-education and support programmes. Peer-support programmes launched during the early 2000s, such as the BC Persons with AIDS “buddy program” (#31), were staffed primarily by volunteers who provided one on one support to PLHIV and their families in understanding treatment options and accessing health care benefits. Recognizing that patients accessing these programmes had complex social and economic needs beyond medical care, AIDS Vancouver staff shifted towards what is now known as a case-management model, in which social workers – with the support of volunteers – assist clients in addressing other needs such as housing and employment [53,54]. This model has since been taken up and institutionalized within regional health services and is a key part of the HIV response today.

### Structural

Between 2000 and 2002, BC adopted a major policy shift away from one-for-one needle exchange policies – which were shown to limit access to sufficient numbers of sterile syringes – and towards needs-based distribution models first pioneered by VANDU’s syringe distribution programme [36,55]. Adopted first in Vancouver and then scaled-up provincially, policy changes entailed increasing and diversifying the number of sites distributing syringes, removing limits on numbers of syringes obtained and the requirement to exchange used syringes (#28, 34) [56]. The BC CDC assumed central responsibility for the distribution of harm reduction supplies in 2004 (#54), further increasing harm reduction supplies and condoms available at no cost to approved sites (Figure 3) [57]. These policy changes have been associated with declines in rates of HIV risk behaviour and HIV incidence among injection drug users [56].

**Figure 4.**
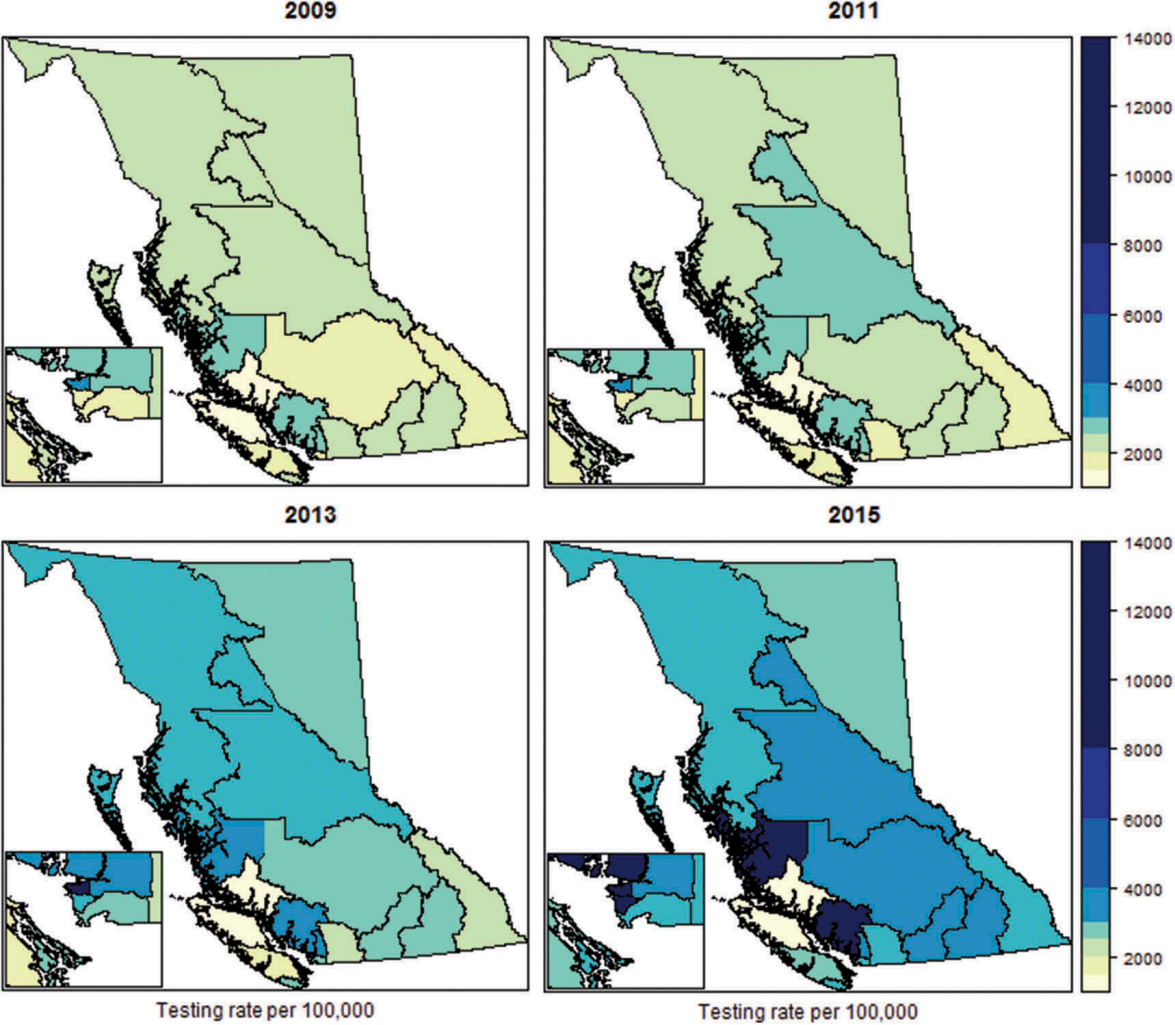
Heat map of HIV testing rates, per 100,000, by health service delivery area, 2009–2015. All testing rates reflect non-prenatal tests. Source: BC Centre for Disease Control.

## The early treatment as prevention phase: 2006–2009

### Biomedical and health services

During the mid-to-late 2000s, HIV cases continued to steadily fall in BC while HAART uptake grew (Figure 1). New evidence suggested these trends may be related, specifically that HAART had a secondary benefit of preventing HIV transmission through suppression of viral load [5,58–60]. In light of these findings, the BC-CfE advocated for a “Treatment as Prevention” strategy to end the epidemic, based on the notion that providing access to HAART at the earliest point following an HIV diagnosis could dramatically reduce the spread of HIV (#54) [61]. This approach would come to form the foundation of a “seek and treat” provincial HIV strategy.

Growth in HAART uptake and viral suppression during this period may be partly attributable to more tolerable and effective HAART regimens, including effective antiretroviral agents for treatment of multidrug resistance HIV [62–67], and the first ever one-pill-daily regimen in 2006 (#85) [68]. The BC-CfE introduced two new initiatives in 2008 to otherwise enhance monitoring of HAART adherence, safety and retention. The Pharmacovigilance Programme was launched to monitor adverse reactions to antiretrovirals, alerting health care providers and patients regarding safety concerns (#92). The HAART interruption alert system, also implemented that year, notifies clinicians if their patients failed to refill their prescription after 60 days (#93).

Despite universal coverage for HAART health care and medication costs, HAART uptake and adherence remained low among many vulnerable populations, with only half of diagnosed PLHIV on HAART and only 68.7% of those on HAART achieving virological suppression (Figure 1). Gaps along the continuum of HIV care at this time were profound among Indigenous PWID, who were twice as likely to become HIV infected compared to other PWID [69], but half as likely to start HAART within the first two years following diagnosis [70]. In Northern BC, home to many Indigenous communities, Positive Living North began delivering HIV prevention and support workshops in 2005 with the goal of better supporting Indigenous PLHIV (#65). The Vancouver Native Health Society launched an HIV treatment support programme in 2007 (#86) and an HIV self-management programme in 2009 (#96) with the goal of better engaging and retaining Indigenous PLHIV in care.

To address gaps in care experienced by female sex workers, local sex work agencies collaborated with public health stakeholders in the mid-2000s to identify structural barriers to care and develop new models of care for female sex workers [71]. These initiatives included integration of a Vancouver drop-in clinic within a support centre for sex workers (#94), HIV prevention and education workshops at a drop-in centre for sex workers in Prince George (#67), and a HAART uptake and adherence programme designed by and for sex workers who use illicit drugs (#88). In Vancouver, collaboration between public health and sex workers eventually evolved into a community-based prospective cohort study that provides voluntary HIV/HCV/STI testing to former and current sex workers(#112) [72].

### Community

While HIV diagnoses continued to decline in the province, these declines were not occurring as quickly among MSM who continued to compose the majority of new cases in BC (Figure 2) [3]. Seeking to mobilize leadership around gay men’s health, the “Health Initiative for Men” (HiM) formed in 2008 from a collaboration of researchers and community members attending the BC Gay Men’s Summit [53]. Based in BC’s lower mainland, the organization works to strengthen the health and wellbeing of gay men by providing MSM-specific health services through their five clinics (#99) and community-based campaigns to raise awareness about HIV, testing and care services. (#100–102,135,172,173,175) [73]. In addition to administering approximately 10,800 HIV tests in 2015/16 alone, HiM is now the widest distributor of condoms to MSM in BC [73].

### Structural

Despite a large body of scientific evidence documenting its public health value [50,74–76], Vancouver’s supervised injection facility faced years of opposition from the Conservative Canadian federal government in power from 2006 to 2015. In 2006, the proposal to extend the drug law exception permitting facility was deferred by the federal Health Minister, effectively cutting funding for research [77]. In response, The Portland Hotel Society, VANDU, and two Insite users filed a statement of claim in B.C. Supreme Court in August 2007 to keep the injection site open. When brought to the Supreme Court of Canada in 2011, all nine judges ruled that attempts to close Insite were unconstitutional (#136) [78]. In addition to allowing Insite to continue operating, the decision paved the way for other jurisdictions in Canada to implement and evaluate similar harm-reduction services.

## The STOP HIV/AIDS phase: 2010-present

### Biomedical and health services

By the early 2010s, mounting evidence suggested that optimal antiretroviral therapy use was a highly effective means of preventing HIV/AIDS morbidity, mortality, and transmission [7,8,79,80]. This evidence-base led the IAS-USA to recommend, for the first time in their 2012 guidelines, that HAART be initiated for all PLHIV, regardless of CD4 count (#137) [81].

Also in 2010, the BC-CfE lab developed an innovative new bioinformatics tool that expedited HIV drug resistance genotyping by automating sequence analysis for HIV drug resistance genotyping (#113). Routine and efficient resistance genotypic testing in turn opened up the possibility of identifying and targeting emerging HIV clusters through phylogentic monitoring. Since 2012, the BC-CfE has implemented a pilot phylogenetic monitoring system that is integrated with comprehensive population-level HIV databases in order to monitor HIV transmission (#142). Monthly and quarterly reports of the growth and characteristic of clusters have been distributed by the BC- CfE since Feb 2014 to the BC CDC and Ministry of Health – where they are reviewed by public health and lab staff to inform public health follow-up [82].

In 2010, the BC Ministry of Health launched the “Seek and Treat for Optimal Prevention of HIV/AIDS” (STOP HIV/AIDS) pilot programme in Vancouver and Northern BC, two regions with a high burden of HIV (#103). The pilot increased funding for HIV testing and treatment, targeted funds for public health intervention, and introduced new surveillance infrastructure to monitor and assess the uptake of HIV-positive individuals into each stage of a continuum of care [19,83].

In Vancouver, pilot activities included establishment of an interdisciplinary outreach team focused on improving HIV care engagement through targeted HIV testing (#146, 125 146), public health follow-up services (#126), service provider training (#122) and intensive case management (#108). To further support testing scale-up, administrators of acute care hospitals in Vancouver recommended offering HIV tests as part of all medical admissions and Emergency Department visiting(#127) [83].

In its pilot programme, the Northern Health authority allocated funds towards launching HIV101.ca, a website that provides comprehensive information on HIV/AIDS testing, treatment and support services in Northern BC (#145). To expand reach of testing in rural settings, Northern Health funded an HIV education and training programme to support communities in implementing HIV testing programmes (#129). Other initiatives include a Medication Adherence Support programme (#117) and expanded Point-of-Care testing at a needle exchange and other designated sites (#100).

Also with funding from STOP HIV/AIDS project, the BC CDC launched a Provincial Point-of-Care HIV testing programme in April 2011 to further expand HIV testing services (#118). Originally launched as a pilot project, the point-of-care HIV programme has since expanded HIV testing to 80 sites across BC where point-of-care tests are made available at no cost to eligible sites, including public health clinics, addictions and mental health sites, outreach programmes, and other community sites [84]. Beyond scaling up HIV testing and care sites, provincial partners sought to improve the quality of care itself through a provincial collaborative launched in 2011 that brought together 17 teams across the province to engage in continuous quality improvement and adult learning activities(#111). This collaborative later (2013) morphed into the HIV quality improvement network, mandated to address gaps in the provincial cascade of HIV care [85].

Given promising initial results from pilot sites, the provincial government decided in December 2012 to implement STOP HIV/AIDS province-wide, making “Treatment as Prevention” the new operating standard in all five of BC’s Health Authorities (#151) [83]. To monitor and evaluate programme outcomes, a cohort of all persons identified as having HIV and living in BC was established alongside the initiative, entailing ongoing linkage of administrative, testing and treatment databases. The resulting STOP HIV/AIDS database has facilitated comprehensive monitoring and assessment of HIV-positive individuals’ engagement along a continuum of care, from initial testing and linkage to care, to HAART initiation and subsequent plasma viral load (pVL) response [13,86]. Mandated with evaluating the provincial strategy, the BC-CfE releases quarterly monitoring reports of these indicators at regional and provincial levels to inform programmatic planning across the five Health Authorities [86].

BC CDC testing data suggests that scale up of STOP HIV/AIDS testing initiatives since 2009 has been associated with increases in HIV testing (Figure 4). HIV testing rates more than doubled province wide between 2009 and 2015. Expansion in HIV testing uptake has been highest in Vancouver, where testing rates nearly tripled from 4125 per 100,000 in 2009 to 11,602 per 100,000 in 2015 [21]. During this period, point-of-care testing was expanded to non-traditional settings – including street fairs, addictions services, bathhouses and community centres, including those in rural and resource-limited settings [87] and among at-risk populations [84].

### Community

A number of targeted social-marketing campaigns were implemented from 2012 to 2014 (#100–102,133–135,161,146,173,175) to complement and promote HIV testing initiatives. One of the widest reaching of these campaigns was “It’s Different Now” (#133), a social and print media campaign developed in Vancouver to prepare the general public for integration of routine HIV testing in family practice. Other campaigns adopted more targeted messaging to reach at-risk populations such as residents of the Downtown Eastside (#46) and MSM (#100–102,135, 161,173, 175).

STOP HIV/AIDS funding provided additional support for peer navigation services providing peer-support and information to PLHIV. In the early 2010s, peer navigation programmes were introduced in a number of sites (#105,113,119,132,167,170), including a newly created HIV/AIDS community centre in Abbotsford (#105) and a chronic health navigation programme in Kamloops. (#110)

### Structural

The shift to a Treatment as Prevention approach was supported by simultaneous changes to BC’s therapeutic and testing guidelines, which recommended from 2012 onward that HAART regimens be prescribed to all PLHIV (#137). HIV testing guidelines released by the BC Centre for Disease Control in 2011 removed the requirement for pre-test counselling before an HIV test, making informed consent the pre-test standard, as it is for other Sexually Transmitted Infections (#120). Provincial HIV testing guidelines released in 2014 further aimed to scale-up HIV testing by recommending annual HIV testing for patients in high-risk populations and testing every five years for all others (#154).

## Discussion

We have provided a historical summary of HIV prevention and care initiatives implemented in BC from the discovery of HAART in 1996 to 2015. Advances in HAART treatment and diagnostics have dramatically improved health outcomes among PLHIV in BC. New HIV cases have declined since HAART was introduced in the mid-90s, despite increases in HIV testing and growth in other STIs (syphilis and gonorrhoea) during the same period [88]. HAART scale up has been advanced through expansion in HIV testing and treatment services and the work of community groups to support individuals in navigating these services. As has been documented elsewhere [12,53], many peer navigation, case management, support services and harm reduction programmes now incorporated into public health programming were first developed by community-based organizations.

BC’s progress in preventing HIV and promoting engagement along the care cascade has in turn been contingent on structural factors. First, BC is unique in the North American context for having a single organization, the BC-CfE, which is centrally responsible for the distribution of antiretroviral drugs and therapy monitoring at no cost to patients [26], as well as clinical education, therapeutic guidelines and population-level outcome monitoring. Provincial investment in HAART expansion has proven to be cost effective in BC, and will likely be cost saving in the long run when considering savings to the health system from averted HIV infections [41]. The use of personal health numbers for all BC-residents allows for rigorous evaluation of HIV strategies through anonymized linkage of HAART drug treatment data to other health administrative datasets. This population-level data has helped identify populations that may need additional support and informing HIV strategies that address the unique epidemics across BC’s various regions.

Second, BC has adopted progressive policies towards harm reduction, which have facilitated wide distribution of harm reduction supplies and otherwise increased opioid agonist treatment access to individuals with opioid dependence (Figure 3). Expansion of OAT and needle distribution since 1996 played a vital role in preventing HIV transmission among PWID, and declines in HIV among PWID has been credited as the primary driver behind consistent declines in BC’s HIV incidence [10].

Third, BC has pioneered a “Treatment as Prevention” approach since 2009 that represents a paradigm shift in HIV testing and treatment by emphasizing routine provider-initiated testing for all British Columbians coupled with targeted initiatives to engage the most marginalized PLHIV in antiretroviral treatment. The provincial strategy has entailed extensive collaboration between the regional health authorities and other provincial partners to expand HIV testing through novel approaches, including point-of-care testing in community settings, integration of HIV care into addictions services, outreach teams, and routine testing of all adults accessing acute and primary care services.

### Limitations

Although we implemented an extensive search strategy to identify interventions, this review only captures those initiatives documented in publicly available grey literature, internal BC-CfE documents and peer-reviewed literature. As such, we may have missed interventions for which there is no evaluation, publication or other publicly available record. The review focuses predominantly on the HIV response in Vancouver, where the HIV epidemic has been largely concentrated and better documented in the literature. While representatives from health authorities provided programmatic information, knowledge about interventions prior to the STOP HIV/AIDS pilot was limited by staff turnover.

The HIV intervention timeline constructed from this review will inform future empirical evaluations of interventions implemented in BC, including a federally funded economic modelling study to identify the optimal combination of strategies, given available resources, to enhance the HIV care cascade within BC’s health authorities. However, such an evaluation is beyond the scope of this paper and figures presented in this manuscript should not be interpreted as implying a causal relationship between interventions and changes in the epidemic.

### Emerging priorities in the HIV response

Despite the successes of B.C’s “Treatment as Prevention” approach, challenges remain in achieving the full potential of HAART, as many PLHIV continue to experience late diagnosis, suboptimal linkage to and retention in care. and other barriers to care [89]. Universal health insurance coverage is not synonymous with universal availability and access to care, given persistent social and geographic disparities observed along the cascade of care [72]. MSM still comprise the majority (58%) of new HIV diagnoses in BC (Figure 2), and HIV prevalence among MSM is estimated to be as high as 18% in Vancouver [90]. Indigenous communities continue to experience a disproportionate burden of HIV and AIDS in BC due to multiple historic and systemic inequities. During the period of 2005–2014, Indigenous people represented 5% of the total provincial population, yet accounted for 11–17% of new HIV diagnoses in BC [2]. This disparity is most profound amongst Indigenous women who make up only 3.5% of the female population in BC, yet comprise approximately one third of all women diagnosed with HIV in 2014 [2].

A devastating rise in provincial overdose death rates – from 4.7 per 100,000 in 2010 to 32.5 per 100,000 in 2016 – has underscored the need for regional scale-up of harm reduction and addiction treatment services. Recent political changes have expedited efforts to scale-up SIF, yet access to harm reduction services continue to be limited in many municipalities due to community resistance and restrictive zoning [91]. Fostering local support and funding for effective harm reduction services will be critical to sustaining and expanding reach of harm reduction services in areas of need.

Further impediments to HIV prevention and treatment implementation exist in the legal arena, as Canada has become one of the most active nations in the criminalization of HIV non-disclosure [92]. The escalating use of criminal law to prosecute HIV non-disclosure raises concerns about the implications on public health efforts to prevent HIV transmission, including the law’s potential negative effect on HIV stigma and HIV testing [93–95].

With widespread, multi-sectoral collaboration, sophisticated surveillance systems to track health system performance, and provincial investment in evidence-based approaches, BC is well positioned to achieve UNAIDS targets of 90% PLHIV diagnosed, 90% on treatment and 90% virally supressed [96]. However sustained, coordinated efforts at each level of the socio-ecological framework and at each stage of the cascade of HIV care will be needed to reach the ultimate goal of ending the HIV epidemic in BC.

## Notes

To access the supplementary material to this article please see Supplementary Files under Article Tools online.
